# In Vitro and In Vivo Toxicological Evaluation of *Avicennia africana* P: Beauv. (Avicenniaceae) Leaf Extract in a Rat Model

**DOI:** 10.1155/2022/3434383

**Published:** 2022-11-07

**Authors:** Mustapha A. Ahmed, Elvis O. Ameyaw, Francis Ackah-Armah, Desmond O. Acheampong, Peter K. Gathumbi, Michael B. Adinortey, George Ghartey-Kwansah, Hope R. Otsyina, Christian K. Adokoh

**Affiliations:** ^1^Department of Biomedical Sciences, School of Allied Health Sciences, University of Cape Coast, Cape Coast, Ghana; ^2^Small Animal Teaching Hospital, SVM, CBAS, University of Ghana, Legon, Accra, Ghana; ^3^School of Pharmacy and Pharmaceutical Sciences, University of Cape Coast, Cape Coast, Ghana; ^4^Department of Veterinary Pathology Microbiology and Parasitology, FVM, University of Nairobi, Nairobi, Kenya; ^5^Department of Biochemistry, School of Biological Sciences, University of Cape Coast, Cape Coast, Ghana; ^6^Department of Forensic Sciences, School of Biological Science, University of Cape Coast, Cape Coast, Ghana

## Abstract

*Avicennia africana* is an important ethnomedicinal plant that has long been used to treat malaria and several other diseases. Despite the plant's antimalarial and other therapeutic properties, there is limited evidence-based data on its potential toxicity. Hence, the purpose of the current study was to assess the safety of *A. africana* leaf ethanolic extract (AAE). The study was designed to ascertain the cytotoxic effects of the crude extract on red blood cells (RBCs) as well as the acute and subacute toxicity in Wistar albino rats in accordance with Organization for Economic Co-operation and Development (OECD) guidelines “Test No. 423” and CPMW/SWP/1042/99. The pulverized, shade-dried plant leaves were sequentially macerated with 70% ethanol to obtain the crude extract (AAE). The extract's cytotoxic activity (CC_50_) against the uninfected human red blood cells (RBCs) was determined using the 3-(4,5-Dimethylythiazol-2-yl)-2,5-diphenyl tetrazolium bromide (MTT) assay. For the acute toxicity studies, the rats (male and female) were divided randomly into six groups of five rats (*n* = 5) and dosed orally once with the following dose levels: 100, 300, 1000, 3000, and 5000 mgkg^−1^, p.o. of the extracted AAE, with the control group receiving only the vehicle. In the repeated dose toxicity studies, the rats (both sexes) were orally administered daily with AAE at 100, 300, and 1000 mgkg^−1^ for 14 days. Rat body weights were measured, and blood samples were tested for haematological and biochemical markers. Internal organs like the heart, kidney, liver, and spleen were collected, inspected, and weighed, and histological examinations were performed. The median lethal dose (LD_50_) value is greater than 5000 mgkg^−1^ body weight, with no significant change in bodyweight or relative organ weight (ROWs) of the extract-treated groups or control group. The extract showed greater cytotoxicity activity (CC_50_), which was >100 *μ*g/mL, compared to the reference drug (artesunate).The dosage groups of 100 and 300 mgkg^−1^﻿bwt had neutrophilia and lymphocytopenia (*p* < 0.05). However, changes in these haematological parameters may not be dose dependent and could be stress related. All the serum biochemical markers studied in rats given AAE did not show any significant change (*p* > 0.05). Histopathological examination of internal organs of AAE-treated rats did not show any significant abnormalities resulting from the extract treatment compared to the control group. Based on the findings in the present study, the LD50 value of AAE was found to exceed 5000 mgkg^−1^ in the acute toxicity test, while the no observed adverse effect level (NOAEL) in rats was 1000 mgkg^−1^ p.o. In the sub-acute toxicity tests. Histopathological analysis revealed no morphological abnormalities in the vital organs.

## 1. Introduction

Despite the availability of orthodox medications in Ghana, about 70% to 75% of Ghanaians are inclined to the use of plant-based medicine for general healthcare maintenance [1]. The reliance on these natural products may be due to their availability, perceived efficacy, and safety as local medicinal plant preparations [2]. Medicinal plants have been suggested to have tremendous therapeutic potential over the years, and when exploited successfully, they could be incorporated into the traditional health care system [3]. Ghana's tropical forest cover is said to contain around 21,000 plant species. Most of these plants possess therapeutic properties and are utilized across the country to treat various diseases [4]. The mangrove cover also provides useful resources and offers a multipurpose livelihood support system to mangrove dwellers.

*Avicennia africana* is a member of the Avicenniaceae family and one of the eight noteworthy species of the genus Avicennia. The plant grows along the shore in areas of seabed, streams, estuaries, and rivers, and has a world-wide distribution [5]. Many ethnomedicinal uses of various parts of the mangrove plant have been proposed. In folklore, the plant is used to remedy a variety of ailments, including cancer, asthma, and diabetes, as well as rheumatism, ringworm, lice, mange, and ulcers. Additionally, the plant is reported to treat some kinds of skin parasites [5], including malaria [6]. The plant's usage by mangrove dwellers was allegedly attributed to a presumed potency and safety arising from a long folkloric history of use [7]. Plant-based medicines have long been thought to be safer than synthetic drugs because they are natural products [8]. This idea has been around for a long time, amid scant evidence-based research on the safety of plant-based medicines. Research has demonstrated that many medicinal plants are innately toxic because of their components and can result in inauspicious reactions if erroneously used [9]. To allow for rational discussion and clear up any doubt concerning the safety of the medicinal plants, it's anticipated that toxicity investigations may reflect their traditional use. Even though the plant is widely used for the treatment of several diseases, its toxicity has not been scientifically tested. Hence, the aim of this research was to evaluate the toxic effects of 70% ethanolic leaf extract of *A*. *africana* using *in vitro* and *in vivo* assays.

## 2. Materials and Methods

### 2.1. Plants Collection, Identification, Preparation, and Extraction

The plants (leaves) were collected from a mangrove forest near Elmina, Cape Coast in Ghana's Central Region. The field is located between latitude N 5° 5 55.122 north of the equator and longitude W 1° 19 22.277 west of the Greenwich Meridian, and it stretches along the University of Cape Coast's coastal boundary. A botanist identified and authenticated the plant, and it was given the voucher specimen number CC3096 for future reference. The specimen was stored in the herbarium of the University of Cape Coast's Department of Environmental Science, School of Biological Sciences. The leaves were meticulously washed and dried under shade at room temperature. Afterwards, the dried leaves were pulverized into a powder form. The pulverized plant material (900 g) was macerated with ethanol (70%, v/v) for 72 h [10]. The filtrate was concentratedusing a rotary evaporator (R-114 SABITA) at 40 rpm and 40°C. The resulting extract was stored in a desiccator at room temperature. The residue of the plant material was remacerated to increase the extract yield. The weight of 178.06 g (19.78%) of the crude extract was obtained and stored at −20°C in a freezer until used.

### 2.2. Animal Care and Treatment

To evaluate the plant's toxicological effects *in vivo*, Wistar albino rats of either sex with a weight range of 151–216 g and aged 7 to 8 weeks were utilized in the experiment. The rats were obtained from the University of Ghana Medical School's Animal Unit in Korle-Bu, Accra, Ghana. They were housed in metal cages and given commercially prepared rat food, acquired from Big Stars Ghana Ltd., as well as water daily. Before the experiment, the animals had to get used to the lab environment (acclimatization) for at least 7 days.

### 2.3. Cytotoxic Impact of A. africana Leaf Extract (AAE) on RBCs

The cytotoxic effects of AAE on erythrocytes were assessed using a slightly modified version of the 3-(4,5-dimethylthiazol-2-yl)-5-diphenyltetrazolium bromide-MTT assay (Sigma Chemical Company, St. Louis, USA) as described by Ayisi et al. [9]. One hundred microliters (100 *μ*L) of crude AAE in a two-fold serial dilution (100–6.25 *μ*g/mL) was deposited into distinct wells of a 96-well microtiter plate in triplicate. Following that, each well received 100 *μ*L of uninfected erythrocytes. Artesunate served as the positive control drug. Subsequently, the cultures were incubated at 37°C for 72 h in an atmosphere of 5% O_2_ and CO_2_. After that, twenty microliters (20 *μ*L) of MTT assay (7.5 mg/mL PBS) were dispensed into the wells, and the mixture was incubated for another 4 h. About 150 *μ*L aliquot of the culture media was carefully removed and discarded from each of the wells. The plates were then treated with acidified isopropanol (200 *μ*L) containing 1% Triton X-100, mixed very well, and stored under dark conditions for 24 h at ambient temperature to facilitate dissolution of formazan crystals. A plate reader was used to measure the optical density of the plates at 570 nm (Tecan Infinite M200, Austria). To determine the concentration of AAE that will cause the death/destruction of half of the red blood cell population (CC_50_ values), the calculated percentage mean cell viability/RBC survival in the AAE-treated wells was plotted against the concentrations of the extract with the aid of Microsoft Excel 2013 software. Using the dose-response curves, we were able to extrapolate the concentrations of crude extract and artesunate that were most likely to cause cell death at 50% of the dose [11].

### 2.4. Acute Oral Toxicity Study

Thirty (30) healthy Wistar albino rats (weighing 151–216 g and aged 7–8 weeks, either sex) were used and grouped into six (6), with five animals in each. The animals were then marked and kept in separate cages for an additional seven days to acclimate to laboratory conditions before extract administration. Each rat in each of the five groups received a single dose of the extract (AAE) orally. The doses ranged from 100, 300, 1000, 3000, and 5000 mgkg^−1^, with one group serving as a control (1–2 mL/100 g distilled water). As AAE was sticky, a few drops of 2% tween 20 were used to emulsify and dissolve in distilled water. The average weight of the rats in each cage was used to calculate the dosage plan for oral administration, and individual rats were dosed based on their weight. Cage-side monitoring and observation were performed to check for mortality and signs of toxic effects in the first 30 min and then hourly for the next 8 hours and the first 24 hours post-AAE administration. The rats were observed for an additional 13 days for symptoms of toxicity and death in the animals. The tests for acute toxicity (Test No. 423) were done according to the guidelines of the Organization for Economic Co-operation and Development (OECD) [12].

### 2.5. Sub-acute Oral Toxicity Studies

In the sub-acute toxicity study, twenty (20) healthy rats were employed, and the selected animals were grouped as described earlier. The animals in this test were allowed seven days of acclimatization. Food and water were withheld overnight. To aid in dose calculation, the initial weights of the animals in all groups were determined, and each rat in all treatment groups received an extract ranging from 0.84 to 1.1 mL of AAE emulsified with a few drops of 2% tween 20 and dissolved in distilled water. Based on the results of the acute toxicity studies, three dose levels (100, 300, and 1000 mgkg^−1^) were used in the sub-acute studies. This was similar to Wanjiru et al.‘s study with slight modifications [13].

Freshly prepared AAE preparations were administered orally and daily to rats in the treatment group for 14 days [14, 15]. The control group also received (1-2 mL distilled water containing 2% tween 20 p.o ) for the 14-day period. The extract was administered at the same time (8 a.m.) every day. Cage-side observation and monitoring were done as described earlier and at least twice (10 a.m. and 4 p.m.) daily for toxicity signs such as salivation, piloerection, urinary incontinence, gait, tremors, convulsions, morbidity, and mortality.

### 2.6. Haematological Indices

On the 15th day (one day after terminating the treatments), blood was collected (2 mL) via cardiac puncture from each of the rats in all study groups after the animals were given light anaesthesia of pentobarbitone (50 mgkg^−1^, i.p). To examine the effects of AAE on haematological parameters, one millilitre (1 mL) of blood was quickly put into one-millilitre vacutainers containing K_2_EDTA anticoagulant and mixed well with a roll mixer. The remaining 1 mL of the blood sample was put into a gel-separated vacutainer to obtain serum. The whole blood samples were analysed using the URIT-5250Vet 5Part-Diff Auto Haematology Analyzer (Urit Medical Electronic Co., Ltd., PR, China) for haematological parameters. The parameters tested include red blood cells (RBC), mean cell haemoglobin (MCH), packed cell volume (PCV), mean cell volume (MCV), haemoglobin (HGB), red cell distribution width (RDW), and mean cell haemoglobin concentration (MCHC). The rest of the parameters that were analysed are: white blood cells (WBC), lymphocytes (LYM), neutrophils (NEU), monocytes (MON), eosinophils (EOS), basophils (BASO), platelets (PLT), mean platelet volume (MPV), and platelet distribution width (PDW).

### 2.7. Biochemical Parameters

The blood sampled in gel-separator vacutainers was given enough time (1 h) to coagulate. The tubes were centrifuged for five minutes at 3000 rpm (Centurion Scientific, UK) to get the serum. The sera were stored at −20°C before being analysed for biochemical markers with the URIT-8021AVet Automated Chemistry Analyzer (Urit Medical Electronic Co., Ltd., SN 8021Avet81000, PR, China). The blood chemistry variables measured were total bilirubin (TB), globulin (GLB), total protein (TP), albumin (ALB), indirect bilirubin (IDB), direct bilirubin (DB), creatinine (CRE), urea, and alkaline phosphatase (ALP). Also, parameters tested in addition to the above were total cholesterol (T-Chol), alanine aminotransferase (ALT), aspartate aminotransferase (AST), triglycerides (TG), high-density lipoprotein cholesterol (HDL-C), low-density lipoprotein cholesterol (LDL-C), very low-density lipoprotein cholesterol (VLDL-C), and calcium (Ca^2+^).

### 2.8. Relative Organ Weight

The rats were killed in a humane way by dislocating their cervical spines, and the body weights were recorded. Following a necropsy, the heart, kidneys, liver, and spleen were inspected, isolated, and weighed. The relative organ weight of each rat was determined as described earlier by both Rajeh et al. and Sharma et al. The formula for calculating relative organ weight in this study is given as follows[14, 16]:

(1) $$\begin{array}{c} \text{Relativeorganweight}\left(\text{ROW}\right)=\frac{\text{Absoluteorganweight}}{\text{Bodyweight}}\times 100\%\text{.} \end{array}$$

### 2.9. Histopathology of Vital Organs

Each rat's vital organs were harvested, grossly examined for lesions, and preserved in formalin (10% v/v). The organs were trimmed and placed in tissue cassettes for routine histopathology using a series of graded ethanol and xylene. The processed tissues were then embedded in paraffin wax blocks. A semiautomated rotary microtome was used to cut sections of 5.0 *μ*m in thickness from the blocks. Sections were mounted on slides and stained with haematoxylin and eosin (H&E) [14]. A light microscope (Olympus, Japan) with a digital camera (AmScop 3.7 digital camera, MD500, USA) was used to examine the histological slides for lesions and other signs of toxicity. For a more detailed presentation, photomicrographs at x400 magnification were taken.

### 2.10. Data Analysis

The data was presented as the mean and standard error of the mean (S.E.M). A percentage mean RBC survival was calculated, and a graph was plotted for the determination of CC_50_ from dose-response curves using Microsoft Excel 2013. The haematological and biochemical results were computed by means of GraphPad Prism, version 7.00 (GraphPad Software, San Diego, CA, USA). One-way ANOVA was used to evaluate if there were significant differences in mean value between the control and experimental groups. The statistical significance between the treatment and control groups was attained via Tukey's post-hoc test. The margin of error was set at 5%.

## 3. Results and Discussion

### 3.1. In Vitro Studies

#### 3.1.1. The Cytotoxic Effect of AAE on Human RBCs

**Figure 1:**
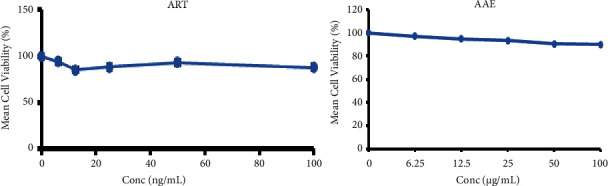
Erythrocytes' survival after exposure to the crude extract of *A. africana* (AAE) and artesunate (ART). Cytotoxic effects (CC_50_ values) of AAE and ART are greater than 100 *μ*g/mL.

Preclinical toxicological studies on all drugs, particularly plant-based medications, permit the collection of data regarding the safety and quality of drugs using *in vitro* as well as *in vivo* inquiries [17]. The current study evaluated the cytotoxicity and acute as well as sub-acute toxicity profiles of *Avicennia africana* extract (AAE) to establish preliminary safety data that can be used to corroborate or refute claims of safety from repeated folkloric applications. The cytotoxicity of the extract was evaluated on uninfected erythrocytes alongside the artesunate drug in the MTT assay. In this study, the cytotoxicity activity of AAE wasdetermined as the cytotoxicconcentration (CC_50_) >100 *μ*g/mL. The positive control (artesunate) also registered a CC_50_ value of >100 *μ*g/mL. In this preliminary study, the cytotoxic effects of the crude extract were like those of the positive control, which showed that more RBCs survived. This is shown in Figure 1.

The use of cold maceration in this work was inspired by prior research on the plant. The plant was reported to have a high concentration of highly polar compounds such as alkaloids, tannins, saponins, reducing sugars, and flavonoids [18], which was confirmed by Ahmed [19].

An *in vitro* bioassay was deployed in this study to predict the presence of a potentially toxic compounds in the extract [20]. In the current study, the cytotoxicity of AAE was first assessed using human erythrocytes in an MTT assay to ascertainthe toxicity at the cellular level. The *in vitro* cytotoxicity test showed that both the extract and the control had negligible cytotoxicity on the erythrocytes. The cytotoxic concentration of AAE required to kill 50% of uninfected red blood cells was not determined in the present study, indicating high erythrocyte survival outcomes in both the extract and artesunate. The RBC survival values suggest that AAE and artesunate have negligible toxic effects on erythrocytes in the MTT assay. This assay was chosen for the determination of the cytotoxicity because it can transform the yellowish tetrazolium to formazan by some types of viable enzymes in erythrocytes [9]. The conversion is made possible subject to the extracts' ability to maintain the RBCs at their normal morphologies, which is also linked to the extracts' intrinsic bioactive elements that protect the cells from cell-mediated degradation. The outcome of the cytotoxicity activity of AAE and artesunate from this assay demonstrated that AAE has inherent natural ingredients that preserve human erythrocytes against exogenous-mediated damage [21]. These results set the stage for acute and subacute investigations, with the predictable possibility that this plant may be of low toxicity.

### 3.2. In Vivo Studies

#### 3.2.1. Acute and Subacute Oral Toxicity Study

In the acute toxicity tests, it was found that AAE did not cause any morbidity or mortality in the animals at doses up to 5000 mgkg^−1^ [22]. The rats did not exhibit any toxic-related symptoms at a dose level of 5000 mgkg^−1^ p.o. In the acute toxicity test after being given AAE. Within 24 h and even up to 72 h after extract administration, no respiratory distress, aggression, diarrhoea, salivation, vomiting, or mortality were observed. There were no mortalities registered or observed to have occurred at the dose levels used up to 5000 mgkg^−1^ of AAE. The LD_50_ for *A. africana* crude extract was not determined in this study, suggesting that the LD_50_ of the AAE is greater than 5000 mgkg^−1^. Thus, the oral LD_50_ of AAE appears to be more than 5000 mgkg^−1^, indicating that dosage levels of up to 5000 mgkg^−1^ are tolerable and relatively safe. Our findings agree with those of Tauheed et al., who found that drug candidates with an LD_50_ of 5000 mgkg^−1^ or higher were safe for therapeutic use in acute toxicity studies [23].

In the sub-acute toxicity test, there was no fatality observed after the 14-day daily dose of extract (100–1000 mgkg^−1^ p.o.). Additionally, no physiological or behavioural abnormalities and several other toxicity indications, such as reduced feeding, tremors, or piloerection, were seen in the AAE-treated animals as opposed to the untreated animals.

#### 3.2.2. The Consequences of AAE on Haematological Indices

Measurement of haematological biomarker thresholds forecasts the level of toxicity of the substance [22, 24]. Moreover, haematological indicators in animal models are crucial for assessing toxicity concerns. Thus, any deviation from the normal blood variables in the cardiovascular system has a higher prognostic value for detecting haematotoxicity. The outcome of the haematological test revealed that the red blood cells, white blood cells, monocytes, eosinophils, basophils, platelet count, platelet distribution width, haemoglobin, haematocrit, mean cell volume, mean cell haemoglobin, mean cell haemoglobin concentration, and red cell distribution width parameters in the extract-treated groups showed no substantial difference (*p* > 0.05) from those of the untreated group. In comparison of the haematological biomarkers, there were significant differences between the control group and the rats given 100, 300, and 1000 mgkg^−1^ of the extract. The neutrophils exhibited a significant increase in values (*p* < 0.05) for the AAE-treated animals in the 100 and 300 mgkg^−1^ groups as opposed to the 1000 mg/kg^−1^ and the control group. As demonstrated in Table 1, there was neutrophilia in rats given 100 and 300 mgkg^−1^ AAE (*p* = 0.002), compared with the 1000 mgkg^−1^ AAE-treated animals and the control group. The variation in these parameters may not be dose dependent.

Previous research has shown that the effects of plant extracts on animals and humans in terms of cardiovascular toxicity, haemototoxicity, and gastrointestinal toxicity may be interconnected [25]. It is noteworthy that most of the haematological parameters (Table 1) evaluated in the present investigation had not changed significantly (*p* > 0.05). Although some parameters, such as neutrophils and lymphocytes, showed significant differences in values (*p* < 0.05) among the AAE-treated animals (100 and 300 mgkg^−1^ as opposed to the control group), this outcome may not have been dose dependent since these parameters were normal at the dose level of 1000 mgkg^−1^, which was the highest dose in this study. Plant extracts may cause neutropenia by suppressing growth factors, for example, granulocyte macrophage colony-stimulating factor (GM-CSF) and granulocyte colony-stimulating factor (G-CSF), which regulate neutrophil synthesis and deployment [26]. Neutropenia may be caused by infections due to microbial pathogens such as viruses, bacteria, protozoans, and fungi. Reduced neutrophils may also be triggered by an intrinsic disorder of proliferation and maturation of myeloid and stem cells [27]. Neutrophilia is a characteristic feature of infections or inflammatory reactions [28].

The pharmacological effect of AAE at 100 and 300 mgkg^−1^may have increased GM-CSF production in rats, resulting in an increase in neutrophils. It is possible that the extract has a stimulating effect on the chemokines and cytokines that control their receptors. This would explain both the AAE effect and the increase in neutrophils [28]. Lymphocytopenia, on the other hand, can be caused by an active viral infection, or it could be caused by damage to the thymus or lymphoid architecture, which stops the body from making enough lymphocytes [29].

The neutrophilia and lymphocytopenia (*p* < 0.05) recorded in the 100 and 300 mgkg^−1^ groups may be stress-induced in the animals during animal handling [30, 31]. In the current study, there was no clear dose-response relationship, which might have resulted in the changes in these parameters by the extract that was tested. Overall, the haematological results of this investigation demonstrate that AAE has no significant detrimental impact on blood parameters.

#### 3.2.3. Effect of AAE on Serum Biochemical Parameters

All blood chemistry markers in rats, including aspartate aminotransferase, total proteins, albumin, alkaline phosphatase, alanine transaminase, total bilirubin, high-density lipoprotein, total cholesterol, very low-density lipoprotein, low-density lipoprotein, triglyceride, urea, creatinine, indirect direct bilirubin, and calcium, did not alter substantially (*p* > 0.05) following 14 days of daily AAE administration. There may be slight variations in all the blood chemistry parameters. However, up to a dosage level of 1000 mgkg^−1^, these differences are not statistically significant (Table 2).

This study also found that none of the biochemical parameters that were measured changed at the highest dose level (1000 mgkg^−1^) because all the biomarkers in this study had normal values in all the profiles that were analysed. Since AST, ALT, TB, DB, IDB, TP, ALP, ALB, and GGT levels did not change substantially (*p* > 0.05) in all extract-treated animals when compared to the untreated group, there is sufficient evidence to rule out liver damage (Table 2). Notably, ALT is a particular liver enzyme that, when elevated, indicates hepatocellular injury. However, in predicting liver disease, AST is considered a less specific biomarker. It may be present in other organs but, when elevated, may suggest hepatic injury. Both ALT and AST are expected to rise dramatically in drug-induced toxicity [32]. High levels of ALP have been linked with biliary cirrhosis and obstructive jaundice [33]. Similarly, ALP levels are also pervasively elevated in bile duct obstruction. However, these enzymes did not change much in AAE-treated groups, which means that toxic-induced hepatobiliary disease is unlikely. Furthermore, histopathology excludes liver damage from the findings of this study.

None of the kidney function test results changed substantially (*p* > 0.05) in any of the animals that took AAE. A decrease in glomerular filtration is the clinical manifestation of increased levels of CRE and urea, which are common in injured kidneys. In this study, the extract had no effect on kidney-related parameters like CRE and urea (*p* > 0.05) in animals that were treated with the extract compared with those in the control group (Table 2).

Several studies have linked increased levels of total cholesterol (T-Chol), triglycerides (Trig), and low-density lipoprotein (LDL) to a rising risk of coronary artery disease, cardiovascular disease, and ischemic stroke [34, 35]. In this investigation, there was not enough evidence to suggest toxic-related cardiac damage in the AAE-treated rats. This is because the extract had no significant (*p* > 0.05) effect on any of the biochemical parameters tested.

### 3.3. The Impact of AAE on Body Weight

Alterations in body weight and relative organ weight in rats have been recommended as sensitive markers in toxicological evaluation to identify changes in organs that have been chemically exposed to toxicants [36]. The impact of AAE on rats' body weight after 14 days of extract administration is presented in Table 3. The variations in body weight of rats from pretreatment to postextract treatment were not statistically significant compared with the control animals during the 14-day period.

Body weight changes are also a factor in determining the overall health of laboratory animals and may be influenced by exposure to potentially toxic substances [37]. In the current test, body weights of the extract-treated rats after the 14-day extract administration did not show any significant differences (*p* > 0.05) when compared with the reference or control group (Table 3).

#### 3.3.1. Effect of AAE on Relative Organ Weight (ROW)

Internal organs may undergo a variety of pathological changes because of the direct effects of toxic materials, causing weight changes. Internal organs, including the liver, kidney, heart, lungs, and spleen, are often the first organs to be affected by toxic substance-induced metabolic reactions [38].  Table 4 illustrates the impact of AAE extract on the relative organ weights of rats following fourteen days of daily extract treatment. The vital organs, including the kidneys, heart, spleen, and liver, did not show any significant changes in ROWs as opposed to the untreated group.

The relative organ weights of AAE-treated animals had not changed significantly (*p* > 0.05) from the untreated group in this study (Table 4). Thus, repeated extract doses up to 1000 mgkg^−1^ during the 14-day period did not show any significant variation in organ weight, which means the extract did not harm the internal organs.

### 3.4. Histological Sections of Vital Organs

**Figure 2:**
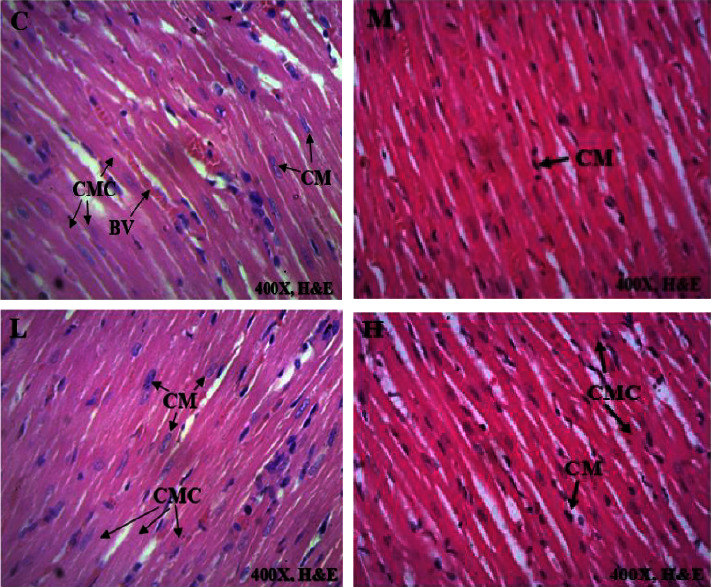
The effect of AAE on histological features of the heart, with (C) representing control, (L) representing 100 mgkg^−1^, (M) representing 300 mgkg^−1^, and (H) representing 1000 mgkg^−1^. CM-cardiac muscle nuclei, CMC-cardiac muscle cells, BV-blood vessels. The photomicrographs (H&E, x400) of the heart tissues represent the general appearance observed in rats treated with AAE and the control group.

**Figure 3:**
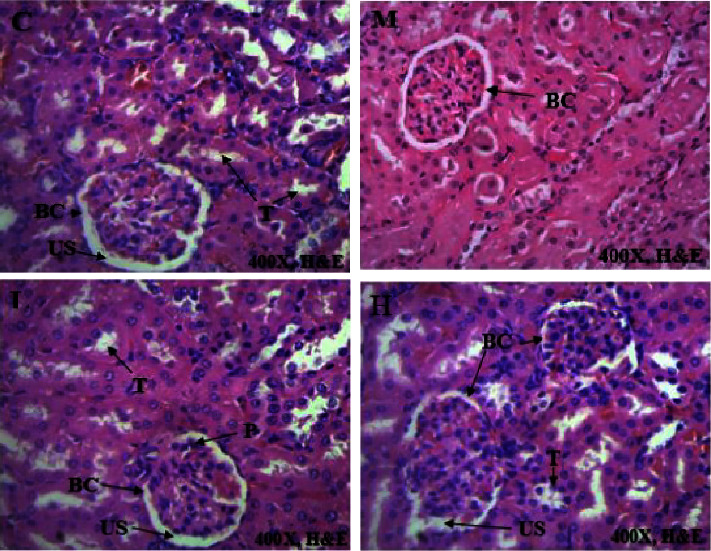
Effect of AAE on histological features of the kidney. C control, L 100 mgkg^−1^, M 300 mgkg^−1^, and H 1000 mgkg^−1^. BC- Bowman's capsule, US- urinary space, T-tubule, and P- nucleus of podocytes. The photomicrographs (H&E, x400) of the kidney tissues represent the general appearance observed in the rats dosed with AAE and the control group.

**Figure 4:**
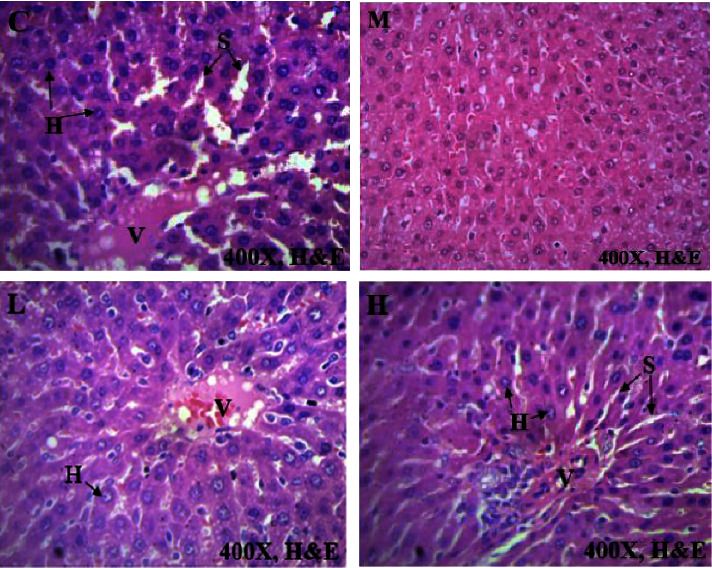
Effect of AAE on histological features of the liver, (C): control, L 100 mgkg^−1^, M 300 mgkg^−1^, and H1000 mgkg^−1^. H-hepatocyte, S-sinusoids, and V-central vein of the lobule. The photomicrographs (H&E, x400) of the liver tissues represent the general appearance observed in the rats treated with AAE and the control group.

**Figure 5:**
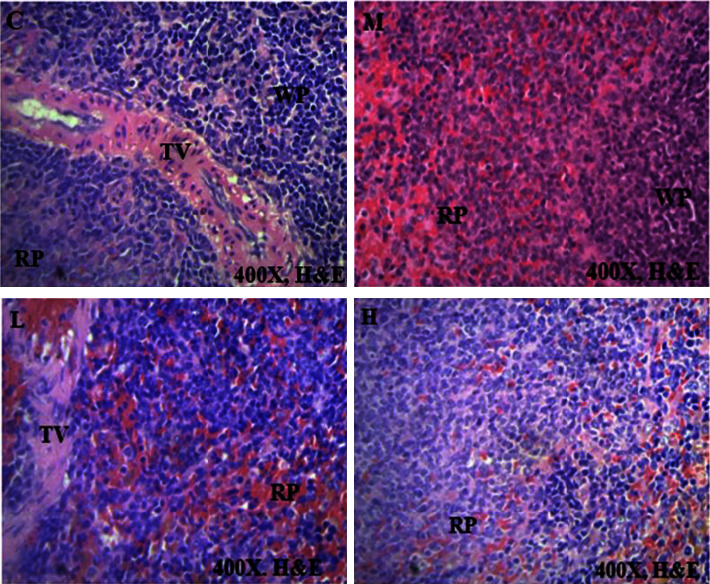
Effect of AAE on histological features of the splenic tissues, **C**: control, **L**: 100 mgkg^−1^, **M**: 300 mgkg^−1^, and **H**: 1000 mgkg^−1^. WP- lymphoid follicles of white pulp, RP- vascular matrix of red pulp, and TV-trabecula vein. The photomicrographs (H&E, x400) of the spleen tissues represent the general appearance observed in the rats treated with AAE and the control group.

Histopathology of selected internal organs was performed to corroborate the haematological and biochemical indices in the current preclinical toxicity studies as outlined in the 2002 OECD guidelines 423 [12, 15]. Macroscopically, no morphological abnormalities were identified in any of the vital organs of the rats dosed with AAE as opposed to the untreated animals. There were no histological abnormalities in the heart, kidney, and spleen, as these vital organs had the same normal architecture as those in the control group (Figures 2–5). Hepatocytes of the liver parenchyma were mostly normal looking, but some had large vesicular nuclei with an abundance of pink cytoplasm. Several sparsely scattered liver cells had dark, compact nuclei with more eosinophilic cytoplasm.

At necropsy, the AAE-treated rats and untreated rats did not have hepatomegaly, hepatic vascular damage, interruption of bile production or flow, or ascites. However, there was a mild hepatocellular degeneration or death (necrosis) in the histopathology evaluation. This is consistent with a single-cell hepatocyte necrosis (death) amid many normal ones in the liver parenchyma. Since most hepatocytes are normal, single-cell necrosis of hepatocytes would have little effect on liver function. However, it has been suggested that until 75% of hepatocytes are dead, liver function does not decline. The liver may be the only solid organ that utilizes regenerative processes to achieve complete recovery [39]. Depending on the degree of damage, liver injury is followed by vigorous hepatocyte multiplication to substitute dead cells and eventually induce spontaneous healing [40]. It is possible that the extract could not have caused the mild single-cell hepatocellular necrosis that was found in all groups of animals, as the same lesions were observed amongst the control animals. Because the changes in liver cells were minor, with a large proportion of the liver parenchyma being normal (Figure 4), they could not have had a significant impact on the overall outcome. The results were consistent with the normal clinical chemistry parameters associated with a normal liver. On the other hand, the heart, kidneys, lung, and spleen had a normal architectural structure in the AAE-treated animals compared with the control group (Figures 2–5).

## 4. Conclusion

The finding shows that *A. africana* crude extract has no harmful effects on red blood cells. The extract demonstrated no evident toxicity in both acute and sub-acute tests, up to a dose level of 5000 mgkg^−1^. The neutrophilia and lymphocytopenia observed in the 100 and 300 mgkg^−1^ animals were not dose dependent and could have been caused by stress. These findings show that *A. africana* is hypothetically nontoxic for oral ingestion at doses up to 1000 mgkg^−1^ for repeated dosing in subacute toxicity studies. Further study is required to evaluate the chronic and subchronic toxicity of the plant extract.

## Figures and Tables

**Table 1:** Haematological indices in animals orally treated with the AAE in the subacute toxicity tests.

Haematological indices (MGKG−^1^)					
Parameters	Control	100	300	1000	*p* value
RBC(M/*μ*L)	8.72 ± 1.15	7.97 ± 0.46	7.84 ± 0.97	7.54 ± 0.12	0.832
HGB (g/dl)	16.10 ± 1.55	13.97 ± 0.68	13.77 ± 1.08	13.65 ± 0.25	0.537
HCT (%)	42.77 ± 4.71	36.97 ± 0.68	39.10 ± 3.98	35.30 ± 0.00	0.532
MCV (FL)	49.40 ± 1.76	46.47 ± 0.86	50.47 ± 4.19	46.90 ± 0.80	0.605
MCH (PG)	18.63 ± 1.09	17.50 ± 0.38	17.73 ± 0.81	18.05 ± 0.65	0.704
MCHC(G/DL)	37.67 ± 0.67	37.67 ± 0.33	35.33 ± 2.40	38.50 ± 0.50	0.270
RDW (%)	13.30 ± 0.24	12.93 ± 0.88	14.07 ± 1.29	12.60 ± 0.50	0.793
PLTX102 (K/*μ*L)	1.27 ± 94.50	1.56 ± 248.80	1.43 ± 200.32	1.69 ± 41.50	0.484
MPV (FL)	4.27 ± 0.15	3.73 ± 0.09	4.30 ± 0.17	3.85 ± 0.25	0.119
PDW (FL)	6.70 ± 0.32	6.80 ± 0.56	5.93 ± 1.07	7.15 ± 0.35	0.481
WBC (K/*μ*L)	6.43 ± 1.25	8.32 ± 0.77	2.99 ± 1.49	8.31 ± 2.50	0.062
LYM (%)	48.55 ± 3.66	25.48 ± 5.21^a,b^	26.91 ± 7.26^a,b^	48.04 ± 8.85	0.031
MON (%)	5.62 ± 1.78	5.09 ± 0.49	4.63 ± 1.58	9.04 ± 0.35	0.082
NEU (%)	41.49 ± 3.27	65.47 ± 5.7^a,b^	64.91 ± 7.63^a,b^	37.51 ± 4.29	0.002
EOS (%)	3.87 ± 0.80	3.73 ± 1.05	1.32 ± 0.56	5.05 ± 4.19	0.563
BASO (%)	0.47 ± 0.07	0.23 ± 0.09	0.20 ± 0.17	0.36 ± 0.02	0.217

One-way ANOVA with Tukey's multiple comparison tests was used to analyze the data. The values represent the mean ± S.E.M. (*n* = 5) (14 days) The letters (**a**) indicate a statistically significant difference between the control and treatment groups; the letters (**b**) indicate a statistically significant difference between the 1000, 100, and 300 mgkg^−1^ groups.

**Table 2:** Serum biochemical indices in animals orally dosed with the AAE in the subacute toxicity tests.

Biochemical Values (mgkg^−1^)					
Parameters	Control	100	300	1000	*p* value
AST (U/L)	92.80 ± 3.66	86.68 ± 11.78	92.90 ± 3.95	96.30 ± 2.40	0.581
ALT (U/L)	50.52 ± 3.88	47.45 ± 2.79	53.51 ± 7.46	44.81 ± 11.41	0.395
ALP (U/L)	36.13 ± 9.57	57.67 ± 4.98	43.67 ± 3.99	55.35 ± 6.45	0.169
TP (g/L)	63.40 ± 4.58	64.63 ± 4.11	65.73 ± 4.00	65.00 ± 2.65	0.217
ALB (g/L)	28.53 ± 1.42	28.83 ± 0.94	31.93 ± 2.78	33.20 ± 0.50	0.378
GLB (g/L)	34.87 ± 3.83	34.67 ± 4.49	38.17 ± 1.93	43.45 ± 2.15	0.502
A/G	0.84 ± 0.10	0.85 ± 0.10	0.84 ± 0.09	0.77 ± 0.03	0.616
TB (*μ*mol/L)	5.87 ± 1.41	3.87 ± 0.26	4.17 ± 0.52	3.35 ± 0.05	0.148
DB (*μ*mol/L)	1.70 ± 0.70	1.80 ± 0.15	1.83 ± 0.29	1.20 ± 0.50	0.792
IDB (*μ*mol/L)	4.17 ± 1.00	2.07 ± 0.28	2.33 ± 0.79	2.15 ± 0.45	0.140
CRE (*μ*mol/L)	80.60 ± 18.41	62.58 ± 5.28	54.40 ± 5.15	65.72 ± 12.25	0.320
Urea (mmol/L)	6.43 ± 0.42	6.30 ± 0.81	5.37 ± 0.97	6.55 ± 0.45	0.856
Ca (mmol/L)	2.33 ± 0.04	2.38 ± 0.03	2.58 ± 0.06	2.37 ± 0.05	0.353
Trig (mmol/L)	0.91 ± 0.08	0.89 ± 0.03	0.87 ± 0.01	1.08 ± 0.13	0.343
Tchol (mmol/L)	1.63 ± 0.14	1.93 ± 0.38	2.03 ± 0.01	1.99 ± 0.08	0.752
HDL_C (mmol/L)	0.63 ± 0.05	0.46 ± 0.24	0.74 ± 0.11	0.90 ± 0.10	0.324
LDL_C (mmol/L)	0.58 ± 0.13	1.06 ± 0.61	0.89 ± 0.12	0.60 ± 0.08	0.792
VLDL_C (mmol/L)	0.42 ± 0.03	0.41 ± 0.01	0.40 ± 0.01	0.49 ± 0.06	0.398

Data analysed using one-way ANOVA with Tukey's multiple comparison test. Values represent mean ± S.E.M. (*n* = 5) (14 days).

**Table 3:** Body weight (g) of animals dosed with the AAE in the sub-acute toxicity tests.

Body weights (g)			
**Doses**	Initial	Final	*p* Value
**Control**	208.87 ± 7.27	239.13 ± 16.62	0.084
**100 mgkg** ^**−1**^	172.04 ± 12.15	193.28 ± 2.86	0.183
**300 mgkg** ^**−1**^	167.51 ± 5.90	208.07 ± 18.61	0.103
**1000 mgkg** ^**−1**^	161.39 ± 2.49	148.14 ± 33.11	0.716

The values are shown as mean ± S.E.M (*n* = 5). Paired *t*-test was used to analyze the data.

**Table 4:** Relative organ weights (ROWs) of animals treated with the AAE in subacute toxicity tests.

Relative organ weight (mgkg^−1^)					
Organ	Control	100	300	1000	*p* value
Liver	2.84 ± 0.30	3.40 ± 0.04	2.98 ± 0.22	3.51 ± 0.08	0.100
Kidney	0.65 ± 0.06	0.66 ± 0.03	0.61 ± 0.03	0.65 ± 0.05	0.526
Heart	0.42 ± 0.05	0.39 ± 0.01	0.43 ± 0.02	0.43 ± 0.01	0.372
Spleen	0.27 ± 0.04	0.35 ± 0.07	0.26 ± 0.02	0.27 ± 0.04	0.150

Organ body indices of AAE-treated rats with the negative control group. The values are given as mean ± S.E.M. (*n* = 5). The data were analysed using one-way ANOVA with (*p* > 0.05).

## Data Availability

The underlying data supporting the results are included in this paper.

## Additional Points

*Highlights*. (i) Traditionally, Avicennia species have been used as medicine for a wide range of diseases, including malaria. (ii) No potential toxicity signs were observed in all rats, demonstrating how safe the extract is. (iii) The 70% ethanolic leaf extract of *A. africana* is potentially safe for oral consumption at doses of up to 1000 mg/kg bwt for repeated dosing.
